# Migration intentions of Asian and African medical students educated in China: a cross-sectional study

**DOI:** 10.1186/s12960-019-0431-z

**Published:** 2019-11-21

**Authors:** Wen Li, Hong Sun

**Affiliations:** School of International Education, Xuzhou Medical University, No. 209 of Tongshan Road, Yunlong District, Xuzhou, Jiangsu China

**Keywords:** Low- and middle-income countries, High-income countries, Health policy, Healthcare workforce, International medical student, Medical migration, Return intention

## Abstract

**Background:**

In recent years, the number of students from Asian and African countries to study medicine in China has been on the rise. This study investigated the migration intentions of China-educated international medical students (IMSs) after graduation and the factors that influence the migration intentions.

**Methods:**

The cross-sectional, questionnaire-based study involved the IMSs from the 2nd to the 6th year of degree course at Xuzhou Medical University, China, conducted from April to July of 2018. The self-administrated questionnaire asked questions on students’ migration destinations for short-term stay and permanent stay. The influence of gender, continent of origin, academic performance, and family socioeconomic background on the migration intentions was analyzed. Chi-square tests were used for statistical analysis.

**Results:**

Among 266 valid responses, 124 (46.62%) students intended to return to their home countries. This intention to return was associated with Asian citizenship, lower academic performance, and middle/lower family socioeconomic status. The remaining 142 students desired to stay temporarily or permanently outside their home countries. The starting time for them to stay outside home countries was immediately after graduation or some time later. Among them, 88 (61.97%) expected to migrate to a high-income country. The intention to migrate to high-income countries was associated with female gender and higher academic grades. For students who intended to stay outside their home countries, the most popular destination for short-term stay was China, and that for permanent stay was the USA.

**Conclusion:**

IMSs with characteristics of Asian citizenship, lower academic performance, or middle/lower family socioeconomic status are more likely to return to their home countries after graduation, and those with characteristics of female gender or higher academic grades are more likely to migrate to high-income countries. These results suggest that China-educated IMSs constitute a potential resource of healthcare workforce not only for their home countries, but also for the recipient countries. Our findings provide important information on healthcare workforce planning for the governments of the relevant countries.

## Background

Many students from Asia and Africa choose to study medicine abroad for a variety of reasons, such as clinical training in a higher-income country [1], seeking education opportunities, and paying low tuition fees [2], facilitated by the international alignment of the medical curriculum and English language [3]. According to reports by the Medical Council of India, nearly 20% of the total Indian medical students choose to study medicine abroad annually [4, 5], and approximately 40% of all the Nepalese medical students studied overseas in 2015 [6]. In African countries, the medical education system is characterized by the small number of medical schools and low student intake [7]. Furthermore, some medical disciplines are not available in many African medical schools. For example, the only medical school in the Union of the Comoros enrolls nursing and obstetrics students, but not other medical disciplines [8]. As a result, many African students are forced to study overseas to pursue their medical career.

For students from lower-income countries, China is one of the most popular destination countries to study medicine [2]. According to the latest statistics, China is now hosting over 68 000 international medical students (IMSs), mostly from Asian and African countries [9]. Some medical schools in China provide a 6-year undergraduate program for IMSs taught in English, which consists of 5-year courses of theoretical and practical studies and 1 year of compulsory clinical rotation internship. At the end of the program, qualified students are awarded a bachelor’s degree in medicine. Study visa is required for IMSs to study in China. For those who intend to work in China after graduation, they are required to apply for a work visa.

China-educated IMSs contribute to the health workforce in their home countries or abroad. Retrospective surveys were conducted by Tianjin Medical University and Guangxi Medical University [10, 11]. The surveys revealed that the majority of international medical graduates returned to their home countries, and the rest stayed in China or migrated to other countries including the UK, the USA, Canada, Australia, and Saudi Arabia. However, these studies did not explore the graduates’ characteristics which influenced their migration choices. Moreover, these studies were conducted more than 7 years ago, and hence, the results may no longer be applicable to the current situation, since the society has been developing rapidly and the whole world has undergone dramatic changes.

Low- and middle-income countries (LMICs) [12] do not have adequate human resources for health [13]. Therefore, the return of China-educated IMSs to their home countries can help alleviate this shortage. On the other hand, high-income countries (HICs) [14] benefit from incoming foreign medical professionals, regarding them as an important source of medical workforce and indispensable primary care providers [15–17]. As China-educated IMSs are potential healthcare workforce for both their home countries and their migrated countries, in the study, we explored their migration intentions after graduation and analyzed the factors associated with the migration intentions.

## Methods

### Setting and questionnaire

This was a cross-sectional, questionnaire-based study conducted between April 2018 and July 2018, at Xuzhou Medical University in China. A self-administered questionnaire was designed according to the purposes of the current study and was distributed to IMSs from the 2nd year to the 6th-year at the university.

The first section of the questionnaire collected students’ basic demographic information, such as name, age, gender, country, and parents’ occupation. The second section asked the students to provide their migration destinations for short-term stay (further education/training or temporary employment), and permanent stay (stable job and life), and to provide reasons for their intentions. The migration intentions were categorized into “home country” and “a foreign country,” and if they chose the latter, they were required to write down the name of that country. The questionnaire was written in the English language as it is the language of instruction for IMSs in this university.

### Data collection and data analysis

All questionnaire forms were distributed to the students in the classroom. Participation by the students was voluntary, and the purpose of the study was fully explained to them prior to the distribution of the questionnaire. The person who distributed the questionnaire was involved in the teaching affairs management of the IMSs at the university and thus was familiar with the IMSs, which might contribute to the high response rate in this study. All the questionnaires were collected on the spot. Phone calls or personal visits were made to those who had not completed the questionnaire.

The academic performance of each student was evaluated with reference to his/her own batch of students (the same year level), because at the time of survey, the 5th- and 6th-year students had completed 63 study subjects, the 4th-year students had finished 45 subjects, while the 3rd-year students and the 2nd-year students had just done 30 and 16 subjects respectively, implying that the average scores would vary among batches. Students with average score ranking above 50% of their own batch were assigned into “above medium” group, and those with average score ranking below 50% of their own batch were assigned into “below medium” group.

Their family socioeconomic status was classified into higher occupations, intermediate occupations, and lower occupations based on their parents’ occupations, and the grouping standard followed Rose and Pevalin’s classification [18], the National Statistics Socio-economic Classification: managerial and professional occupations were grouped into higher occupation; business, teacher, and other white-collar occupations were grouped into intermediate occupation; and lower supervisory, less skilled, and routine task occupations were grouped into lower occupation.

Responses were entered into a database and analyzed using SPSS. Students’ short-term and permanent migration intentions were summarized. Chi-square tests were performed to determine whether gender, continent of origin, academic performance, and family socioeconomic status were associated with the students’ migration intentions to return to their home countries or to go to an HIC.

This study was approved by the Ethics Committee of Xuzhou Medical University.

## Results

### Demographics

Of 301 students who were given the questionnaire, 268 completed the survey, translating to a response rate of 89.04%. The majority (53.36%) of the students were male. Fifty-four (20.15%) responders were the 6th-year students who were about to graduate, 57 (21.27%) were the 5th-year students who had finished their pre-internship course study, 54 (20.15%) were the 4th-year students, 50 (18.65%) were the 3rd-year students, and 53 (19.78%) were the 2nd-year students. In total, 204 (76.12%) were from Asian countries, and among those, 165 were Indian, 20 were Bengalis, and 19 were Nepalese; 64 (23.88%) were from African countries, and among those, 15 were Zambian, 13 were Nigerian, 7 were Ghanaian, and the remaining were from Zimbabwe (6), Somalia (5), Union of the Comoros (4), Ethiopia (3), Democratic Republic of the Congo (3), Tanzania (2), Burundi (1), the Republic of Congo (1), Cameroon (1), Kenya (1), Mozambique (1), and Uganda (1). Data from 2 3rd-year students who had not decided where to go after graduation was excluded from data analysis, so a total of 266 valid responses were analyzed.

### Return intentions

As shown in Table 1, 117 (43.98%) students planned to stay outside their home countries temporarily, and 149 (56.02%) planned to return home for a short-term stay; 78 (29.32%) named a place outside the home country as their permanent destination, while 188 (70.68%) preferred to work and live in their home countries. Overall, 124 (46.62%) students intended to return home (choosing the home country both as the short-term and permanent destinations).

**Table 1:** Students’ migration intentions

Migration intentions	Respondents	%
Return intentions (*n* = 266)		
Students intending to return to the home country ^a^	124	46.62
Students choosing home country as short-term destination	149	56.02
Students choosing home country as permanent destination	188	70.68
Students expressing an interest to stay outside the home country ^b^	142	53.38
Students choosing a foreign country as short-term destination	117	43.98
Students choosing a foreign country as permanent destination	78	29.32
Intentions to migrate to an HIC (*n* = 142)		
Students expressing an interest to go to an HIC ^c^	88	61.97
Students choosing an HIC as short-term destination	56	39.44
Students choosing an HIC as permanent destination	60	42.25
Students intending to go to an LMIC ^d^	54	38.03
Students choosing an LMIC as short-term destination	61	42.96
Students choosing an LMIC as permanent destination	18	12.68

^a^“Students intending to return to the home country” referred to those who chose the home country both as short-term and permanent destinations

^b^“Students expressing an interest to stay outside the home country” referred to those who chose a foreign country either as short-term or permanent destination

^c^“Students expressing an interest to go to an HIC” referred to those who chose an HIC outside the home country either as short-term or permanent destination

^d^“Students intending to go to an LMIC” referred to those who chose an LMIC outside the home country both as short-term and permanent destinations

### Intentions to migrate to an HIC

More than half (142, 53.38%) of the responders expressed that immediately or some time later after completion of the medical study in China, they would stay outside their home countries temporarily or permanently. Further analysis of the country choices in the context of HICs and LMICs revealed that, out of the 142 students, 88 (61.97%) had intentions to migrate to HICs and 54 (38.03%) chose to go to LMICs (Table 1).

### Destination choice outside the home country

Table 2 provides a list of the students’ destination choices outside their home countries. Among all HICs, Canada was the most popular place for short-term stay, and the USA was the most preferred destination for permanent stay. Among all LMICs, China was chosen as the most favorite short-term destination and permanent destination.

**Table 2:** Students’ destination choices outside the home country

Destination country	Short-term stay (*n* = 117)	Permanent stay (*n* = 78)
HIC	56	60
Canada	12	10
Australia	10	12
USA	9	14
UK	7	8
Germany	4	5
Netherlands	3	1
Belgium	2	3
Other HICs or ones not specified	9	7
LMIC	61	18
China	53	12
South Africa	2	1
Other LMICs or ones not specified	6	5

### Influence of students’ characteristics on return intentions

Table 3 shows the characteristics of the students with intentions to return to home countries. Although a higher percentage of males planned to return home compared to females, there was no statistically significant difference between genders in their return intentions (*P* > 0.05). Geographically, a significantly higher percentage of Asian students (56.37%) intended to return to home countries compared to African students (14.52%) (*P* < 0.01). Moreover, the majority of the students with lower academic performance (53.19%) intended to return to home countries compared to those with higher scores (39.20%) (*P* < 0.05). Family socioeconomic status was identified to be a significant factor influencing the participants’ return intentions (*P* < 0.05); pairwise comparison revealed that the proportion of students with higher family socioeconomic status in those expressing intentions to stay outside their home countries was significantly higher than that of their counterparts with middle/lower family socioeconomic status (*P* < 0.05). Therefore, we concluded that students with middle/lower family socioeconomic status were more likely to return to their home countries.

**Table 3:** Students’ characteristics influencing return intentions (*n* = 266)

Characteristics	Students intending to return to the home country	Students expressing an interest to stay outside the home country	*χ*^2^	*P*
Gender				
Male	68 (48.23%)	73 (51.77%)	0.31	0.58
Female	56 (44.80%)	69 (55.20%)		
Continent of origin				
Asia	115 (56.37%)	89 (43.63%)	33.48	< 0.01
Africa	9 (14.52%)	53 (85.48%)		
Academic result				
Above medium	49 (39.20%)	76 (60.80%)	5.21	< 0.05
Below medium	75 (53.19%)	66 (46.81%)		
Socioeconomic status				
Higher occupation	30 (28.85%)	74 (71.15%)	21.78	< 0.01
Intermediate occupation	74 (58.73%)	52 (41.27%)		
Lower occupation	20 (55.56%)	16 (44.44%)		

### Influence of students’ characteristics on intentions to migrate to an HIC

Table 4 shows the characteristics of the students with intentions to migrate to an HIC. There were significantly more female students (76.81%) who chose to migrate to an HIC than their male counterparts (47.95%) (*P* < 0.01). Moreover, students with higher academic grades in those to choose to migrate to an HIC (75.00%) overwhelmingly outnumbered those with lower scores (46.97%) (*P* < 0.05). Continent of origin and family socioeconomic status did not have a significant effect on the students’ plans to migrate to an HIC (*P* > 0.05).

**Table 4:** Students’ characteristics influencing intentions to migrate to an HIC (*n* = 142)

Characteristics	Students expressing an interest to go to an HIC	Students intending to go to an LMIC	*χ*^2^	*P*
Gender				
Male	35 (47.95%)	38 (52.05%)	12.54	< 0.01
Female	53 (76.81%)	16 (23.19%)		
Continent of origin				
Asia	50 (56.18%)	39 (43.82%)	3.39	0.07
Africa	38 (71.70%)	15 (28.30%)		
Academic result				
Above medium	57 (75.00%)	19 (25.00%)	11.78	< 0.05
Below medium	31 (46.97%)	35 (53.03%)		
Socioeconomic status				
Higher occupation	48 (64.86%)	26 (35.14%)	0.61	0.74
Intermediate occupation	31 (59.62%)	21 (40.38%)		
Lower occupation	9 (56.25%)	7 (43.75%)		

## Discussion

In the study, we found about half of the students intended to return to home countries after graduation. Students with characteristics of Asian citizenship, lower academic performance, or middle/lower family socioeconomic status were more likely to return to their home countries after graduation, and those with characteristics of female gender or higher academic rank were more likely to move to HICs. The majority of those students who intended to stay outside their home countries chose China as a destination for short-term stay and the USA as a destination for permanent stay.

Medical students educated abroad are potential healthcare professionals for the countries they migrate into after graduation. Therefore, their migration intentions are important for such countries to develop healthcare workforce policies [19]. However, few studies have investigated the migration patterns of IMSs educated in LMICs, although a number of such studies have already been conducted in HICs [20, 21].

In this study, among China-educated IMSs, 53.38% of the responders showed an interest in staying outside their home countries after graduation. This result is similar to the migration trends of domestically educated medical students in India [22], Bangladesh [23], Nepal [2], Ethiopia [24], and Malawi [25]. Furthermore, only 7% of IMSs educated in Australia intended to return to the home country, while 82% preferred to stay in Australia after graduation [21]. Despite the shortage of medical recruits, the continuous exodus of health workers from LMICs, due to factors such as low payment, poor working conditions, and few career development opportunities, exacerbates the shortage of physicians in these countries, further compromising the healthcare delivery and health outcomes [24, 26].

Our study showed that about 20% of the IMSs chose China as the short-term destination for postgraduate education or further training, but less than 5% chose it as a place for permanent stay. The popularity of China as a short-term destination may be related to IMSs’ undergraduate education experience, abundant postgraduate education opportunities, and low tuition fees [2, 27]. However, because of the insurmountable language barrier [28, 29], which hinders IMSs from effectively communicating in the hospital practice and obtaining the medical license in China [30, 31], many IMSs are reluctant to choose China as a country for practicing medicine.

The results from this study show that return intentions of IMSs did not differ significantly between genders. This finding is consistent with some of the previous studies, exemplified by one study investigating the career plans of Malawian medical students [25], and another looking at the career practice locations of Nepalese physicians [32]. However, there are also other reports presenting a different result. Some researchers found that female health workers from Asia and Africa were more likely to remain in home countries [19, 24], possibly due to factors related to social and family considerations [19, 33]. However, we have not found any previous literature that analyzed gender differences in medical migration intentions to an HIC. In this study, we found that more females chose an HIC than males. Two reasons may contribute to this result. Firstly, highly educated female students are motivated to make the choice due to their ambition and courage to compete for opportunities. Secondly, the conservative societal attitudes towards working women [34] and the inequality in training opportunities as well as other gender disparities in non-pecuniary benefits in some LMICs [35] may also play a role in pushing female IMSs to choose a higher income country to seek employment or training opportunities.

Our study has not only identified an association between better academic performance and the intention to emigrate [36, 37], but also demonstrated that better academic performance influenced the decision to migrate to an HIC. It is reasonable that medical students with stronger abilities aim higher, which is also the case with brighter physicians, who may leave their home countries for better training and more advanced technology [37]. In fact, the exodus of the highest quality medical talents is currently a serious challenge to many LMICs [3], although Khan [29] argued that the migration of the best physicians would not affect the homeland healthcare service quality as much as it did to medical research.

Our research provides insightful findings that may help policymakers in LMICs to develop strategies or programs which preferentially attract overseas-educated medical graduates to return to their home countries. In fact, Sweden has already developed some policies to attract its overseas-educated medical students, such as encouraging them to come back for clinical practice and offering employment programs tailored to this specific population, in order to promote the domestic physician workforce [38].

## Conclusion

In the study, we have found about half of the IMSs intended to return home after graduation, whereas the other half showed interest in staying outside the home country. The results also show that Asian citizenship, lower academic rank, and lower/intermediate family socioeconomic status were factors associated with IMSs’ return intentions, while female gender and higher academic performance were factors that influenced IMSs’ choice for HICs. Governments of the relevant countries should be aware of the inflow of such graduates and consider the need of such graduates when making healthcare workforce plans.

### Limitations

This study may be more representative of the entire group of IMSs in China if the sample size is larger. Furthermore, the attitude of IMSs’ home countries towards the higher education certificates obtained in China and the consequent influence on their return intentions were not analyzed in our study. Therefore, further studies are needed to explore additional factors influencing IMSs’ migration intentions with a larger sample size.

## Data Availability

All data generated or analyzed during this study are included in this published article and its supplementary information files.

## Abbreviations

HIC: High-income country
IMS: International medical student
LMIC: Low- and middle-income country
UK: United Kingdom
USA: United States of America
